# Evolution of foodborne disease surveillance in China: A 32-year journey from monitoring to early warning

**DOI:** 10.1016/j.onehlt.2026.101348

**Published:** 2026-01-31

**Authors:** Zhifang Zhang, Weiwei Chen, Xuejie Liu, Yanqin Deng, Huarong Hong, Shenggen Wu

**Affiliations:** a Fujian Center for Disease Control and Prevention, Fujian Provincial Key Laboratory of Zoonotic Diseases, Fuzhou 350012, China; b Xiamen City Center for Disease Control and Prevention, Xiamen 361021, China; c Fisheries College of Jimei University, Xiamen 361021, China; d National Key Laboratory of Intelligent Tracking and Forecasting for Infectious Diseases, Public Health Emergency Center, Chinese Center for Disease Control and Prevention, Beijing 102206, China

**Keywords:** Foodborne disease surveillance, Food safety, Whole genome sequencing (WGS), *Vibrio parahaemolyticus*, One health

## Abstract

China's foodborne disease (FBD) surveillance system was implemented later than those in most developed countries. However, in the past 32 years, it has undergone improvements: from pilot projects to full coverage; from a passive mode to an active one; from localized development to the integration of international standards; and from a single function to a comprehensive system.

During this process, China's health administrative departments have adjusted their regulatory departments and functions for FBDs in response to evolving epidemiological patterns of FBD. Simultaneously, they have used a phased, step-by-step approach to promote the use of whole genome sequencing (WGS) technology, according to the level of regional economic development, to facilitate FBD traceability. However, the system must be further improved in terms of traceability capabilities, multi-departmental collaboration, and data sharing mechanisms. At various historical stages, FBD outbreaks in China have shown distinct regional characteristics, and the spectrum of common pathogenic bacteria in China differs from those in the Europe Union (EU) and the United States. In China, diseases caused by microorganisms such as *Vibrio parahaemolyticus, Salmonella, Staphylococcus aureus, Bacillus cereus,* and *Echerichia coli* are dominant. Since 2011, the number of FBD outbreaks has increased each year, and has been accompanied by a decline in the case fatality rate, thus reflecting improvements in foodborne detection technology in China. In the future, further integration of advanced technologies such as WGS will be necessary to enhance surveillance sensitivity, strengthen active and targeted surveillance of key populations, and establish a risk warning model tailored to China's dietary characteristics, thereby increasing the effectiveness of FBD prevention and control.

## Highlights

• A thirty-two-year, decadal-level perspective on China's evolving foodborne disease epidemiology.
• China's foodborne disease surveillance system has undergone a paradigm shift from crisis-driven, passive legislation to proactive, source-tracing monitoring.
• Over the past 32 years, *Vibrio parahaemolyticus* has consistently been the leading bacterial cause of foodborne disease outbreaks in China.
• China needs a "One Health" approach to track foodborne risks from farm to fork and in environmental-animal-human reservoirs.

## 1 Introduction

Driven by expanding global trade and emerging new food products, the increasing food safety risks have established foodborne diseases (FBDs) as a critical public health issue worldwide [1]. FBDs are a major cause of mortality and morbidity worldwide, although they are common in developing countries, including China [2,3]. In Africa alone, approximately 91 million people become sick, and 137,000 die annually from FBD [3]. Even in high-income subregions, FBD remains a significant burden. In the high-income European subregions (EUR A, B, and C), this burden is estimated to be in the range of 40–50 disability-adjusted life years (DALYs) per 100,000 population [4]. The global burden of FBD was estimated at 33 million DALYs per year in 2010, which corresponds to a rate of approximately 474 DALYs per 100,000 population [5]. This difference indicates the markedly higher burden borne by low- and middle-income countries globally.

Strengthening national FBD surveillance and burden estimation is fundamental to shaping effective food safety policies and advancing FBD control efforts globally and nationally [6]. Data from FBD surveillance and outbreak investigations can help determine priorities for control measures, policies, and practices, improve food safety, and decrease disease burden [7]. Surveillance of FBDs enables timely outbreak detection and containment, thereby decreasing transmission risks and safeguarding public health. Quantitative assessment of health and economic effects supports evidence-based policy making to achieve more effective interventions. Therefore, strengthening the development of the national FBD surveillance system is imperative.

In 1995, the World Trade Organization (WTO) emphasized that food safety should not be seen as a simple binary outcome—”safe” versus “unsafe”. Instead, it should be viewed as a continuum of risk [8]. The architecture of the FBD surveillance system critically influences the comprehension of the disease spectrum and its evolving trends. In 2024, the WHO issued international guidelines. These provide a common framework for countries to implement risk-based food inspections. The guidelines recommend building models using relevant data. They also advise periodic adjustments to these models to achieve continuous improvement [9]. Developed countries worldwide have established mature FBD surveillance systems. The U.S. CDC and PulseNet USA are assisting global PulseNet international partners in applying whole genome sequencing (WGS) methods in their laboratories to enable molecular traceability of pathogens and facilitate early outbreak warning [10]. For comprehensive, standardized international comparisons, the European Centre for Disease Prevention and Control (ECDC) Surveillance Atlas is the best tool. The EU uses its integrated Rapid Alert System for Food and Feed (RASFF) platform to consolidate surveillance data from member states, thereby providing the data foundation to support risk assessments by EFSA [11]. Australia regularly releases quarterly and annual reports on FBD surveillance and outbreak investigations based on OzFoodNet monitoring data [12]. Together, these systems exemplify public health surveillance best practices by facilitating international comparison, enabling cross-agency data integration, and thereby ensuring open data sharing and public reporting. China is the world's largest developing country and food consumer market. This scale, combined with distinctive national conditions, including diverse dietary practices and significant heterogeneity in climate, geography, and economic development, presents unique challenges for food safety governance [13]. Consequently, an effective FBD surveillance system in China requires a design of particularly high precision and adaptability, tailored to these specific complexities rather than merely replicating models from industrialized countries.

The present analysis traces the 32-year evolution (1992–2023) of China's FBD surveillance system, documenting its transition from an event-driven, passive framework to a proactive surveillance network. This transformation has been characterized by the progressive refinement of legislation and sustained enhancement of technical capabilities. Unlike analyses confined to annual reporting, this work examines long-term, phase-specific shifts in epidemiological characteristics across three decades. It further explores the underlying drivers behind the persistent high prevalence of high-risk pathogens like *Vibrio parahaemolyticus* and *Salmonella*, including environmental change, dietary habits, and animal production. By quantifying trends in six major foodborne pathogens, the study integrates human health surveillance with food safety, animal, and environmental data, thereby establishing an integrated “One Health” perspective for risk analysis.

## 2 Materials and methods

### 2.1 Data sources

Data were obtained from two sources: (1) relevant documents and data released by the National Health Commission of the People's Republic of China, China National Center for Food Safety Risk Assessment (CFSA), and National Institute for Nutrition and Health, Chinese Center for Disease Control and Prevention (NINH, China CDC); and (2) literature in the China National Knowledge Infrastructure (http://over-sea.cnki.net/kns55/default.aspx), Wanfang Database (http://www.wanfangdata.com.cn/), and Web of Science databases, which were searched for published articles regarding FBD outbreaks in China during 1992–2023. Annual counts of FBD outbreaks, associated morbidity and mortality, etiological agents, and the six most prevalent bacterial pathogens implicated in the outbreaks were extracted. The data for this study were sourced exclusively from aggregated and anonymized surveillance data published in the official reports of Chinese national health authorities and peer-reviewed publications. As no individual-level data were accessed or used, this analysis did not require separate ethical approval.

### 2.2 Reporting criteria

During the 1992–2010 period, the majority of reported food poisoning incidents were larger-scale events, defined as those with over 30 cases. In 2011, the Ministry of Health (MOH) modified the origin food poisoning reporting system to a new national foodborne disease reporting system. Formed by CDCs at all levels, the new network requires the verification and reporting of all foodborne outbreaks consisting of two or more cases (instead of the previous threshold of 30 cases).

### 2.3 Data analysis

The population data were derived from the 2010 Sixth National Population Census of China. Given that the CSFA was established in 2011, the study period from1992 to 2023 was divided into three segments to encompass its inception:1992–2001, 2002–2010, and 2011–2023. Data were input into Microsoft Excel and verified by two researchers. All statistical analyses were performed in R (version 4.4.1). A significance level of α = 0.05 (two-sided) was applied to all tests, including chi-square tests for comparing rates between different pathogenic bacteria.

### 2.4 ARIMA time series forecasting

Annual time series data of pathogen-associated incidents and patients from 2011 to 2023 were used for forecasting analysis (Official 2024 national FBD data has not yet been released by the authorities). The Autoregressive Integrated Moving Average (ARIMA) model was applied to forecast trends in the total number of incidents and patients for the period 2024–2026. The optimal order of the ARIMA model (*p, d, q*) was automatically selected using the auto.arima() function from the R package forecast (version 8.21), based on the corrected Akaike Information Criterion (AICc)—a widely accepted criterion for balancing model fit and complexity while penalizing overfitting. Here, *p* represents the order of the autoregressive (AR) component, *d* denotes the number of differencing steps required to achieve stationarity, and *q* is the order of the moving average (MA) component. Residual analysis showed no significant autocorrelation (*P* > 0.05), indicating that the model provided an adequate fit. Subsequently, the validated ARIMA model was used to generate point forecasts and 95% confidence intervals (*CIs*) for the next three years.

## 3 Results

### 3.1 Food safety supervision, prevention, and control system

**Fig. 1:**
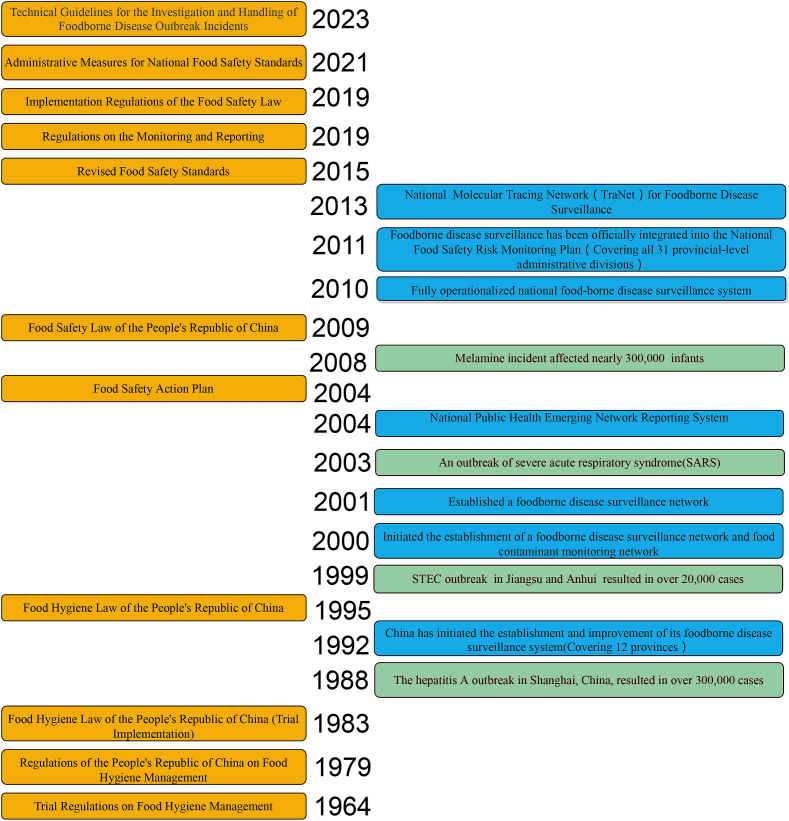
Incident-driven timeline of food safety regulations and surveillance systems in China.

The evolution of China's FBD surveillance system progressed from establishing an initial framework to laying a legal foundation, and was followed by the construction and refinement of the surveillance system, thus ultimately achieving modernization and comprehensive coverage. Throughout this process, three major food poisoning incidents and one major public health emergency have driven revisions to laws and regulations, as well as reforms in the surveillance system, thus continually enhancing China's capacity for FBD monitoring. The hepatitis A outbreak in Shanghai in 1988 infected more than 300,000 people. In 1995, China issued the Food Hygiene Law, which required that food poisoning incidents involving more than 30 cases, or two or more deaths, be reported to local or national governments as emergency events. This law provided a preliminary legal basis for FBD surveillance, thereby marking a new phase in China's food safety management. In response to the SARS outbreak in 2003, the major transformative event in public health in China, an internet-based national direct reporting system was established for public health emergencies in 2004. However, the effectiveness of FBD surveillance has been limited, primarily because laboratory-confirmed data for infectious diarrhea cases are frequently lacking. The 2008 melamine crisis exposed critical flaws in China's food safety regulatory system, thereby prompting comprehensive reforms and enhanced testing capabilities. In 2009, the enactment of the China Food Safety Law explicitly established FBD surveillance as a core component of the national food safety monitoring framework. This legislation marked China's transition from hygiene management to safety governance and indicated substantial progress toward alignment with international food standards. In 2011, the MOH modified the original food poisoning reporting system to a national FBD reporting system. This system required CDCs to report foodborne outbreaks involving two or more cases. According to the Food Safety Law of the People's Republic of China, the Regulations on Public Health Emergencies, and the WHO Guidelines for FBD Outbreak Investigation and Control, the Technical Guidelines for the Determination and Management of FBD were officially promulgated in 2023(Fig. 1). Subsequently, the FBD surveillance system was advanced and gradually aligned with international food regulations.

### 3.2 Development of the FBD surveillance system in China

**Fig. 2:**
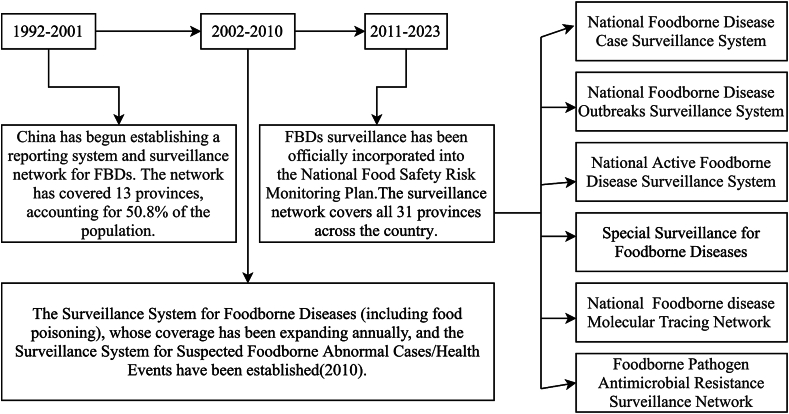
Comparative frameworks of the foodborne disease surveillance system at three key stages (1992–2023).

The development of China's FBD surveillance system can be traced back to 1992, when the monitoring network initially covered 13 Provinces, autonomous regions, and municipalities directly under the central government. In 2000, the NINH established a food poisoning reporting network, which had expanded to cover 21 Provinces by the end of 2008. By 2009, the system had expanded to include 16 Provinces in the food contaminant monitoring network and 21 Provinces in the FBD surveillance network (covering 80% of the population). After the establishment of the CFSA in 2011, the surveillance system was progressively enhanced. The MOH revised the original food poisoning reporting system into the National FBD Reporting System. Under this system, CDCs at all levels are required to verify, investigate, and report incidents involving two or more cases (instead of the previous threshold of 30 cases). The system expanded its coverage from 2854 CDCs (at county level or above) in 29 Provinces in 2011, to 3378 centers across all 31 Provinces by 2016. The current FBD surveillance system includes the National FBD Outbreak Surveillance System (2011) and the National Molecular Tracing Network for Foodborne Disease Surveillance (TraNet, 2013)(Fig. 2). Case surveillance for FBDs encompasses suspected case monitoring, confirmed case monitoring, and cluster case monitoring. In addition, the system conducts targeted analysis of pathogen-food vehicle pairs to pinpoint the most common causative agents in FBD. China has launched specialized surveillance programs for listeriosis, campylobacteriosis and for cronobacter infection in infants and young children in designated Provinces. Provincial-level CDCs are responsible for both pathogen molecular traceability (via pulsed-field gel electrophoresis and WGS) and antimicrobial resistance monitoring (Fig. 2). Parallel to this dedicated FBD surveillance, diarrheal diseases from other foodborne causes are monitored under different programs. National Diarrheal Syndrome Surveillance has also established diarrheal syndrome surveillance sentinel sites, focusing on detecting foodborne pathogens such as non-typhoidal *Salmonella* (NTS), *Shigella*, diarrheagenic *Escherichia coli*, *Yersinia enterocolitica*, *Yersinia pseudotuberculosis*, *Campylobacter jejuni*, *Campylobacter coli*, *Aeromonas*, *Plesiomonas shigelloides*, rotavirus, and norovirus. Additionally, the infectious disease surveillance system includes national sentinel sites for monitoring pathogens that cause enteric infections, such as *Vibrio cholerae*, *Shigella*, *Salmonella Typhi*, Enterohemorrhagic *Escherichia coli*, O157:H7, and *Yersinia enterocolitica.*

### 3.3 Epidemiological trends in FBDs across three periods

**Fig. 3:**
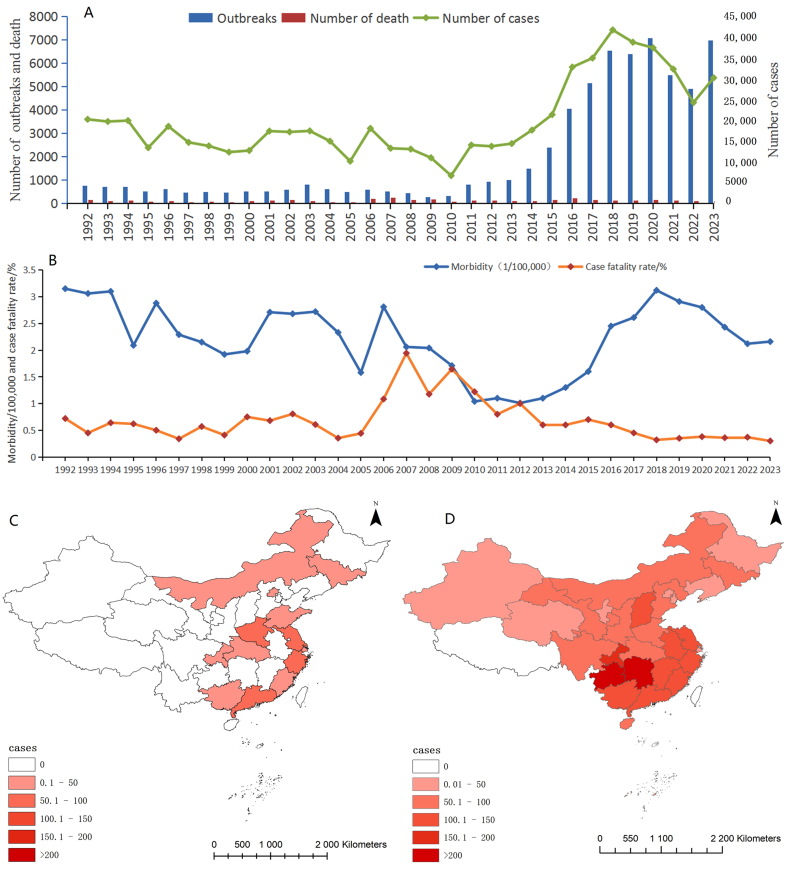
Epidemiological trends in FBDs across three periods in China. A. Number of reported FBD outbreaks, cases, and deaths in 1992–2023. B. Morbidity and case fatality rate in 1992–2023. C. Average annual incidence of FBD outbreaks in China, 1992–2001; D. Average annual incidence of FBD outbreaks in China, 211–2023. The combination of data gaps in 2002, 2007, and 2009, along with the progressive expansion of surveillance regions, precluded the use of interpolation for missing values, as such a method could lead to a misleading interpretation of the underlying trends. As a result, no geographic distribution map for the disease from 2002 to 2010 was generated.

On the basis of the scope of coverage of China's FBD outbreak surveillance and the time of establishment of the relevant national competent authorities, the period from 1992 to 2023 was divided into three distinct historical phases, each exhibiting unique outbreak characteristics. The surveillance network covered 13 Provinces in 1992–2001 (Guangdong Province and Hubei Province lacked data on pathogenic factors and implicated foods). A total of 162,995 cases and 932 deaths were reported. Expanded coverage (14 Provinces in 2005, 18 Provinces in 2006, and 21 Provinces in 2008) led to reporting of 97,486 cases and 731 deaths in 2002–2010. This period included the largest proportion of years with case fatality rates exceeding 1%. Following its expansion to 31 Provinces (autonomous regions/municipalities), the surveillance system reported a total of 353,821 cases and 1670 deaths from 2011 to 2023 (Fig. 3A). To characterize the epidemiological profile of the FBD, we analyzed both temporal trends and spatial patterns. Annual morbidity and case fatality rates over the study period are presented in Fig. 3B, while the geographical heterogeneity of cases across China is depicted in Fig. 3C and D. Over the past 13 years, the reported incidence rate of FBD in China increased annually from 2011 to 2017, followed by a downward trend from 2018 to 2023. In contrast, the case fatality rate has consistently decreased since 2012. The highest incidence of FBD outbreaks in China is concentrated in the of Yunnan Province, Shandong Province, and Guizhou Province.

### 3.4 Etiological variations in FBDs across the three periods

**Fig. 4:**
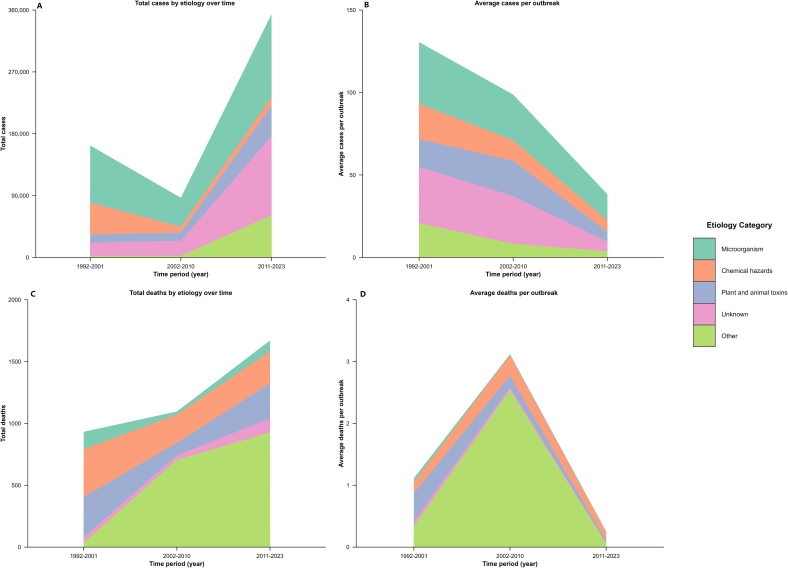
Epidemiological characteristics of foodborne disease incidents across three periods. A. Total cases by etiology; B. Average cases per outbreak; C. Total deaths by etiology; D. Average deaths per outbreak.

During the historical periods of 1992–2001 and 2002–2010, microbial pathogens were the leading cause of both FBD outbreaks and cases. Chemical factors caused the most deaths in 1992–2001, whereas “other” factors were the leading cause of mortality during 2002–2010. From 2011 to 2023, outbreaks of unknown etiology accounted for the most incidents and cases, and other factors were the primary causes of deaths. Because the number of monitored provinces varied in each period, the overall incidence data are not directly comparable. However, during 2011–2023, a slight upward trend in the annual number of FBD cases was observed (Table 1, Fig. 4A, C). Although outbreaks involving toxic mushrooms (others) exhibited the highest case-fatality rate, those caused by microbial and unknown agents led to the greatest number of illnesses per event (Fig. 4B and D).

**Table 1:** Outbreaks, cases, and deaths due to foodborne diseases caused by various pathogenic factors in China across three periods.

Type	Year	Microorganism	Chemical hazards	Animal and plant toxins	Unknown	Others⁎	Total	χ2(*P*)
Outbreaks	1992–2001	2221	2165	715	568	101	5770	15,144(<0.05)
	2002–2010	1522	687	554	760	278	3801	
	2011–2023	7639	1812	7340	19,863	16,507	53,161	
Cases	1992–2001	82,888	46,558	12,040	19,368	2101	162,955	125,439(<0.05)
	2002–2010	42,164	8533	11,882	21,911	2317	86,807	
	2011–2023	120,624	12,735	44,033	115,120	61,309	353,821	
Deaths	1992–2001	141	386	326	45	34	932	991.69(<0.05)
	2002–2010	25	228	103	34	706	1096	
	2011–2023	87	258	285	113	927	1670	

⁎ Including poisonous mushrooms, other fungi and their toxins, and mixed factors., etc.

### 3.5 Top six bacteria causing foodborne outbreaks across three periods

The top six bacterial pathogen responsible for foodborne outbreaks in China are *V. parahaemolyticus, Salmonella, Staphylococcus aureus (S. aureus), Bacillus cereus (B. cereus), Echerichia coli (E. coli),* and *Proteus vulgaris (P. vulgaris).* Over the past 32 years, *V. parahaemolyticus* has caused the most outbreaks, ranking first across all three historical periods, and has been followed by *Salmonella* (Table 2). To determine whether the distribution of FBD outbreaks caused by these six bacterial pathogens differed across the three historical periods, a chi-square test was performed. The results indicated a statistically significant shift in the proportional composition of outbreaks attributable to these six major pathogens (χ^2^ = 644.85, *P* < 0.05). From 2011 to 2023, *V. parahaemolyticus* and *Salmonella* together accounted for 54.6% of microbiologically caused outbreaks, whereas the proportion of outbreaks caused by *P. vulgaris* among all microbiological outbreaks gradually declined.

**Table 2:** Top six bacterial foodborne pathogens by number and proportion of outbreaks, 1992–2023.

Year	*V. parahaemolyticus*	*Salmonella*	*S. aureus*	*B. cereus*	*E. coli*	*P. vulgaris*	Others⁎	Total
1992–2001	623(28.1)	359(16.2)	178(8.0)	136(6.1)	112(5.0)	280(12.6)	533(24.0)	2221
2002–2010	451(29.6)	186(12.2)	157(10.3)	155(10.2)	69(4.5)	173(11.4)	331(21.7)	1522
2011–2023	2188(28.6)	1988(26.0)	864(11.3)	510(6.7)	446(5.8)	187(2.4)	1456(19.1)	7639

⁎ Including *Klebsiella pneumoniae, Burkholderia gladioli, Listeria monocytogenes*, *Shigella, Aeromonas,* Other bacteria, etc.

### 3.6 Retrospective and predictive analysis of six foodborne pathogen

**Fig. 5:**
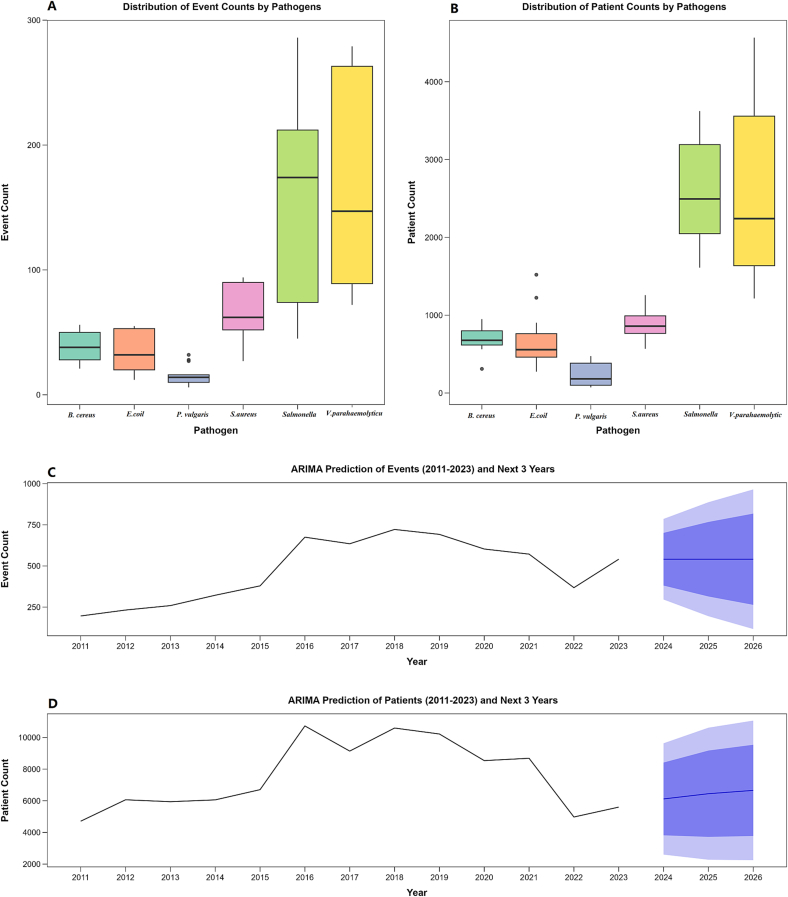
*V. parahaemolyticus, Salmonella, S. aureus, B. cereus, E. coli* and *P. vulgaris* associated incident and case distributions with ARIMA forecasting (2011–2026). A. Distribution of event counts by six pathogens in 2011–2023; B. Distribution of patients counts by six pathogens in 2011–2023; C. ARIMA modeling and 3-year forecast of the combined total number of events for all six pathogens (2011−2023); D. ARIMA modeling and 3-year forecast of the combined total number of patients for all six pathogens (2011–2023).

Between 2011 and 2023, the number of FBD outbreaks caused by 6 types of bacteria increased steadily until 2019, followed by a significant decline in 2022. In terms of case numbers, the trend also rose initially and then fell, with a notable drop between 2019 and 2022, before a slight rebound in 2023. *V. parahaemolyticus* and *Salmonella* caused the highest number of outbreaks and cases. However, both the outbreak frequency and case numbers fluctuated widely across years, showing large variations. This indicates that *V. parahaemolyticus* and *Salmonella* poses a greater food safety risk than the other four bacteria (Fig. 5.). Based on 2011–2023 epidemiological data of six bacteria, this study built an ARIMA model to forecast future disease trends. The initial data showed strong fluctuations, confirmed by the Augmented Dickey-Fuller (ADF) test. After comparing AIC and BIC values, ARIMA (1,1,0) was selected as the optimal model for predicting outbreak numbers. The model's standardized residuals constituted a white noise sequence (Ljung-Box Q test: *P* = 0.8254), demonstrating good predictive performance. For case numbers, ARIMA (0,1,1) also proved optimal (*P* = 0.7123). The established model was used to forecast incidence trends for the next three years (2024–2026). The results point to a stable outbreak scale during this period. The annual number of outbreaks is expected to remain stable. In contrast, the annual number of cases is predicted to show an upward trend. By 2026, the expected number of outbreaks is 541 (95% *CI*: 118–964), while the number of cases is expected to rise slightly to 6656 (95% *CI*: 2248–11,063).

## 4 Discussion

FBDs pose a major global public health challenge, particularly in densely populated China, where their effects are particularly pronounced [14]. In 2023 alone, 6960 FBD outbreaks were reported in China, including 30,237 cases and 90 deaths [15]. The sustained increase in reported FBD cases in China is fundamentally driven by the maturation of the national surveillance system, characterized by comprehensive coverage and enhanced capacity. This evolution, propelled by innovations in molecular diagnostics, has transitioned surveillance from clinical diagnosis to molecular traceability. The resulting gains in detection sensitivity and systematicity have enabled the identification of previously elusive sporadic cases and outbreaks, thereby revealing a more accurate disease burden that is directly reflected in the rising case reports.

The 32-year evolution of China's foodborne disease surveillance system demonstrates that major food safety incidents have frequently served as primary driving forces for advancing surveillance infrastructure and refining legislation, thereby establishing a development model characterized by “Crisis-Driven Response, Systemic Strengthening, and Capacity Enhancement.”Beyond China, the development of modern food safety systems has often been catalyzed by major outbreaks. The 1993 U.S. *E. coli* outbreak catalyzed PulseNet [16]. The FBD surveillance system transitioned to digital online reporting in 2011 and was integrated into the BEAM Dashboard in 2024, enabling multi-pathogen visualization and completing the transition from a paper-based system to an intelligent surveillance platform [17]. The 1996 BSE crisis in Europe spurred the establishment of the European Food Safety Authority (EFSA) [18]. The 2011 German *E. coli* outbreak validated the EU's rapid alert network [19]. And now, the EU FBD surveillance network is a globally recognized, supranational system underpinned by cross-border cooperation and integrated governance. While China's system has converged with those of industrialized countries in key principles, critical gaps remain in surveillance sensitivity, traceability precision, and international collaboration when benchmarked against top-tier standards.

The evolution of China's FBD surveillance system has been driven by two pivotal advancements. The first is the dramatic expansion of surveillance coverage, which grew from 13 to 31 Provincial-level regions and the Xinjiang Production and Construction Corps, thereby significantly increasing case detection. The second is the dramatic acceleration of diagnostic capabilities, exemplified by the shift from daily to hourly reporting, which is shifting the paradigm from passive trace-back to active prevention [20]. Previous research has indicated that the rise in reported cases between 2003 and 2017 was partly attributable to the implementation of mandatory surveillance and improvements in traceability technologies [21]. The optimization of the surveillance network, coupled with the widespread adoption of molecular detection technologies centered on real-time PCR, has improved pathogen confirmation rates, with an average positive detection rate of 11% among diarrheal outpatients from 2013 to 2016 [13]. The extensive application of molecular methods such as PCR has enhanced the efficiency and timeliness of detecting hard-to-culture bacteria. Building upon the foundation laid by PFGE within the TraNet network [22], WGS has now been implemented for FBD surveillance in 29 of China's provinces, marking a transformative shift in the national surveillance system. By providing comprehensive genetic data for accurate pathogen identification and strain differentiation, WGS revolutionizes outbreak detection and investigation. This capability enables earlier discovery of outbreaks, including those previously undetectable, and significantly accelerating source tracking. As a result, WGS is now recognized as an essential tool by public health authorities worldwide [23], and as of 2024, its adoption across these provinces has established it as a key measure for enabling proactive public health responses. In 2025, China has established a nationwide molecular traceability network for FBD based on WGS, utilizing the technical framework provided by the “Management Protocol for the National Foodborne Disease Molecular Traceability Network (TraNet)”. This framework has enabled the system to become the first in China to achieve practical application spanning the national, provincial, and municipal levels. The landscape of FBD surveillance is being transformed by technological revolutions. While WGS has revolutionized outbreak detection and investigation, CRISPR-based assays offer revolutionary speed for on-site detection [24]. Furthermore, non-culture-based methods, such as the application of metagenomic next-generation sequencing (mNGS), are increasingly vital for rapid pathogen identification [25]. These advances, however, generate vast amounts of disparate genetic data. Therefore, to fully harness their combined potential, establishing a national WGS database integrated into global networks becomes a critical step [23]. Such interoperability is essential to unify these data streams, ultimately enhancing the capacity for faster and more precise traceback.

The epidemiological characteristics of FBD outbreaks varied across historical periods, while a consistent pattern of critical regional disparities persisted throughout all eras. Both geographical environment and socioeconomic factors influence the occurrence of FBDs, and environmental factors are the primary driver of the FBD burden in less developed and developing countries [5]. Mushroom poisoning is a common and highly fatal FBD in southwest China, with 925 deaths recorded from 2001 to 2013 [26]. In China, the distribution of FBDs exhibits a south-north declining gradient centered along the Yangtze River axis, water-related contamination and transmission routes are becoming increasingly critical to FBDs. Coastal and riverine regions may emerge as high-burden zones necessitating enhanced governmental prevention and control measures. When studying the drivers of FBD, population density plays a more significant role in western China than in eastern China [27]. Climate variability, such as long-term changes in temperature, humidity, rainfall patterns, and extreme weather, affects food safety throughout the food chain, including during farming, and can also affect food nutritional quality by influencing the occurrence and intensity of FBDs, particularly foodborne diarrheal diseases [28]. The association between climate change and foodborne diarrheal diseases highlights the need to establish a climate-resilient food safety system to decrease FBD transmission [29].

Microbial pathogens are the leading cause of FBD outbreaks that result in major morbidity and socioeconomic costs. Identifying the most important sources and transmission routes of foodborne pathogens is crucial for formulating food safety strategies at the national and regional levels [30]. In recent decades, the main pathogenic factors responsible for FBD outbreaks in China have been microbial, among which *V. parahaemolyticus* and *Salmonella* have been the most common pathogenic bacteria [31]. *V. parahaemolyticus*, transmitted through seafood, poses a dual threat to marine ecosystems and human health [[32], [33], [34]]. The rising global incidence of human vibriosis, primarily caused by pathogenic *Vibrio* species such as *V. parahaemolyticus*, is closely linked to increasing seafood consumption. *Vibrio* contamination in fish poses a direct threat to human health, with its distribution showing geographical and climatic variation. This spatial heterogeneity leads to uneven risks of foodborne infection across regions. As detected in freshwater fish, shellfish, and marine fish in China from 2010 to 2019, the positive rate for *V. parahaemolyticus* was 19% [35]. A meta-analysis (2006–2016) indicated that the overall contamination rate of pathogenic bacteria in foods sampled in China was 8.5% (95% *CI*: 8.2–8.7), with *V. parahaemolyticus* representing the most prevalent pathogen at 21.3% (95%*CI:*19.6–23.1) [36]. *V. parahaemolyticus* isolates from the southeastern coast of China revealed that 95.8% of the strains exhibited a strong capacity for biofilm formation. This indicates that the vast majority of these strains possess a remarkable ability to survive and persist on environmental surfaces and food processing equipment, a trait that was positively correlated with tetracycline resistance [37]. Therefore, it is essential to develop targeted prevention strategies that account for local climatic conditions and contamination profiles. Simultaneously, risk management must be strengthened across the entire supply chain, from aquaculture to retail, to effectively reduce human infection [38]. The implementation of both precise strategies and integrated supply chain management critically depends on a foundational capability: the interoperable sharing of surveillance and monitoring data across all relevant departments and sectors.

*Salmonella* causes approximately 1.35 million infections annually in the United States, with nearly one-fifth of these illnesses attributed to chicken products. In comparison, the prevalence of *Salmonella* was 15.8% in raw poulty meat, 10.9% in raw pork in 2010–2019 in China [35]. The annual incidence rate of foodborne *Salmonella* in China is approximately 1295.59 cases per 100,000 [39]. To reduce and prevent poultry-associated salmonellosis, implementing multilayered prevention strategies throughout the entire farm-to-table continuum is essential [40]. WGS serves as a cost-effective approach in One Health surveillance systems for assessing foodborne *Salmonella*, providing multidimensional insights into pathogen characteristics. *Salmonella* from Chinese food-producing animals commonly exhibit multidrug resistance, high virulence potential, and plasmids facilitating antibiotic resistance gene transmission, such as *IncFII(S)*. These findings directly demonstrate the carriage of multiple risk genes in *Salmonella* along the food production chain from animals to meat products, outlining a potential transmission route from farm to fork [41].

Antimicrobial resistance (AMR) in major foodborne pathogens, such as *V. parahaemolyticus* and *Salmonella*, constitutes a critical public health challenge within the One Health paradigm. The AMR in these pathogens undermines gut microbiota homeostasis and elevates the global FBD burden. This challenge is critically exacerbated by the pervasive dissemination of antibiotic-resistant bacteria (ARB) and genes (ARGs) across the continuum from agricultural and aquaculture environments to animal-derived foods and, ultimately, human hosts [42]. Therefore, effectively mitigating AMR-related FBD requires integrated interventions along this entire chain, with priority given to source prevention and control.

## 5 Conclusion

Over the past 32 years, China has built one of the largest FBD surveillance networks in the world. The country has shifted from passive paper reports to active, web-based alerts that cover 31 Provinces. The data indicate that the number of FBD outbreaks showed a gradual upward trend overall after 2011, with a transient decline observed during the 2020–2023 period due to the impact of COVID-19 containment measures. In contrast, the case-fatality rate exhibited a consistent and steady decrease over the same timeframe. In terms of epidemiological characteristics, the causative agents of FBD have undergone some changes. However, *V. parahaemolyticus* remains the most prevalent pathogen. Moreover, China's FBD management strategy must pivot toward a proactive, prevention-oriented system grounded in the One Health framework. This necessitates a integrated approach that tracks risks, such as AMR in *Salmonella*, from farms to forks, and across environmental, animal, and human reservoirs. This “One Health” approach requires two foundational elements: integrated control measures and cross-sectoral data-sharing mechanisms. These pillars are critically enabled and driven by advanced technologies such as WGS. These integrated efforts are expected to establish a more robust foundation for early detection, timely warning, and precise control at the earliest possible point in the transmission chain.

## CRediT authorship contribution statement

**Zhifang Zhang:** Writing – original draft, Visualization, Funding acquisition. **Weiwei Chen:** Methodology, Formal analysis, Data curation. **Xuejie Liu:** Methodology, Formal analysis, Data curation. **Yanqin Deng:** Formal analysis, Data curation. **Huarong Hong:** Formal analysis, Data curation. **Shenggen Wu:** Writing – review & editing, Visualization, Supervision, Methodology, Funding acquisition, Data curation.

## Ethics statement

The authors have no ethical statement to declare. This study utilizes publicly available, anonymized, and macro-level aggregated data. It does not involve any personally identifiable information or experimental interventions on humans or animals. Therefore, approval from an institutional ethics review committee was not required.

## Declaration of competing interest

The authors declare that they have no known competing financial interests or personal relationships that could have appeared to influence the work reported in this paper.

## Data Availability

The data employed in this study are entirely public, aggregated, and anonymized. They were sourced from official government websites and the published academic literature. The complete original datasets can be obtained from the corresponding official agencies upon reasonable request.
